# Broadening and Strengthening Underrepresented Group Inclusion in Immunological Research

**DOI:** 10.3389/fimmu.2020.00465

**Published:** 2020-03-17

**Authors:** Elaine Smolock, Jacques Robert

**Affiliations:** ^1^Department of Microbiology and Immunology, University of Rochester School of Medicine and Dentistry, Rochester, NY, United States; ^2^Center for Professional Development, Graduate Education and Postdoctoral Affairs, University of Rochester School of Medicine and Dentistry, Rochester, NY, United States

**Keywords:** educational program, Diversity & Inclusion, graduate program, career developement, training & development

## Abstract

Promoting diversity across biomedical fields is crucial for building comprehensive and innovative research programs, as well as providing trainees from underrepresented groups (URGs) the ability to establish agency and develop skills in a culturally and structurally supportive environment. Despite this awareness, there is still a lack of students from URGs being trained for independent research careers. The Immunology, Microbiology, and Virology (IMV) graduate program at the University of Rochester School of Medicine and Dentistry (URSMD) has been working for the last 13 years to increase diversity through an NIH funded Post-baccalaureate Research Education Program (PREP). Historically, our program has trained URG scholars in Immunology, but as we have progressed we have embraced the understanding that both the scholars and the institution benefit from expanding the interdisciplinary nature of our program. Over the last 3 years, we have integrated a broader and highly collaborative faculty mentor pool, including representation from Immunology, Microbiology, Virology, Neuroscience, Genetics, Biochemistry, Biophysics, Toxicology, and Biomedical Engineering. This expansion, coupled with changes in our education program, including skill building workshops and cross campus integration with our student diversity groups and the Office of Diversity and Inclusion, has strengthened the competitiveness and success of our cohorts. These improvements are enhancing the diversity of our graduate school, creating a research environment that retains students from URGs in biomedical research. We attribute our success to the interdisciplinary and team-building nature of our pipeline program, as well as the URSMD's initiatives to be a more inclusive and equitable institution.

## Introduction

It is increasingly recognized that a wide range of scientific disciplines across biomedical fields is crucial for building comprehensive, innovative, and diverse research programs (1–3). Perhaps less obvious, the opportunity to choose among a broad area of research specialties is also important to provide individuals from underrepresented groups (URGs) the ability to establish independent research in a culturally and structurally supportive environment (4, 5). However, to date, this aspect of training is insufficiently developed for URG scholars (URGs) (5). This is further evidenced by the paucity of URGs at the faculty level who are conducting biomedical research (6, 7). We present here how the University of Rochester School of Medicine and Dentistry (URSMD) is successfully diversifying and enriching a long-standing Immunology based program designed for post-baccalaureate URGs. We discuss how we restructured our pipeline research education program to be more inclusive of our scholars' scientific interests centered around immunological research and to foster a research environment that increases trainee retention in biomedical research.

## UR-Prep History and Success—Institutional and Workforce Diversity Enhancement

**Figure 1:**
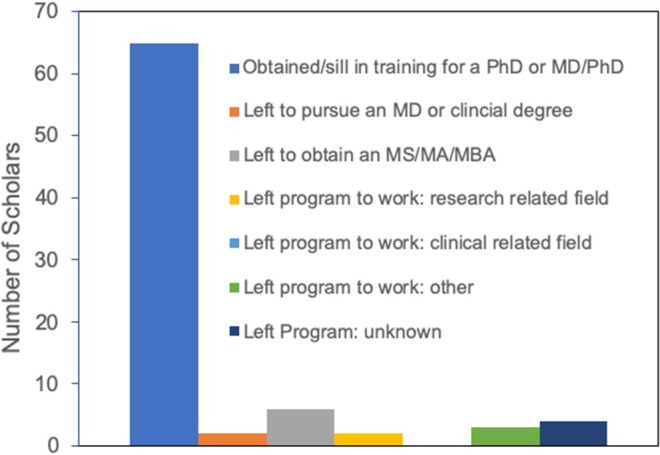
Status of all UR-PREP scholars who entered doctoral training programs (2003–2019).

The URSMD Post-baccalaureate Research Education Program (UR-PREP) was designed to prepare promising URGs to successfully advance in their pursuit of biomedical research toward immunology and infectious diseases. Over the 16-year course of our program (established in 2003 by Stephen Dewhurst, PhD, and succeeded by Edith Lord, PhD) we have successfully trained, or are currently training, 112 scholars. Importantly, 82 (73.2%) scholars entered PhD or dual MD/PhD programs, and 79.2% of these scholars have either graduated with a PhD or are still enrolled in their doctoral training programs (Figure 1). Notably, among all scholars, 74.1% have endeavored to pursue research related careers (Table 1). These data indicate that UR-PREP has been overall successful in its mission to provide URGs the opportunity to develop research and academic skills that will afford them the competencies necessary for graduate school and impactful scientific careers.

**Table 1:** Career/training status of all UR-PREP scholars (2003–2019).

**Current status in biomedical training**	**Percent scholars (%)**
Research Related—Academia	49.1
Research Related—Industry	15.2
Research Related—Government	2.7
Currently applying to Ph.D. Programs	7.1
Clinical	14.3
Other	4.5
Unknown	7.1

While we have witnessed much success, we have come to better understand and appreciate the changing landscape of research interests and that it is imperative to diversify our program. UR-PREP has been rooted in the Department of Microbiology and Immunology, but in 2016 we decided to extend our programming and research experiences across our whole institution, concurrent with implementing a new leadership and organizational strategy. The specific goals were to enhance the research experiences and education of our scholars toward their career goals, to enrich their professional development, and ensure an effective and inclusive mentoring. Data from the literature demonstrate that it is necessary for URGs to develop their skills in a culturally and structurally supportive environment with community support and effective mentoring (4, 8). It is also critical to introduce URGs to the career opportunities afforded to them upon receiving their doctorates (1, 8, 9). Described below are the innovative approaches we have undertaken in the last 3 years of UR-PREP to meet this need, as well as preliminary evidence of our success thus far.

## Approaches to Enhance URGS Inclusion in Immunology Biomedical Research

To better adapt our UR-PREP scholars to the changing immunology landscape, we have markedly restructured our program. First and foremost, we have diversified opportunities to train in immunologically extended disciplines involving a wider range of mentors. This is complemented by a more integrated educational curriculum strongly relying on team spirit and building within the UR-PREP family-like group and across the institution. A synergistic co-director and team leadership approach consolidates the cohesiveness and coordination of this educational program. Notably, our team-based leadership provides UR-PREP scholars easier availability to meet with co-directors, members of the steering committee, or counselors, which enhances sensitivity to any issues they may encounter. In turn, this permits the leadership to better adapt curriculum options and professional development opportunities toward the scholars' needs. This model creates a more attractive research environment, which helps to retain URGs in biomedical research and build a sense of belonging in our URSMD community.

## Extending Research Opportunities From the Immunology Hub

### Diversifying Mentor Involvement

**Figure 2:**
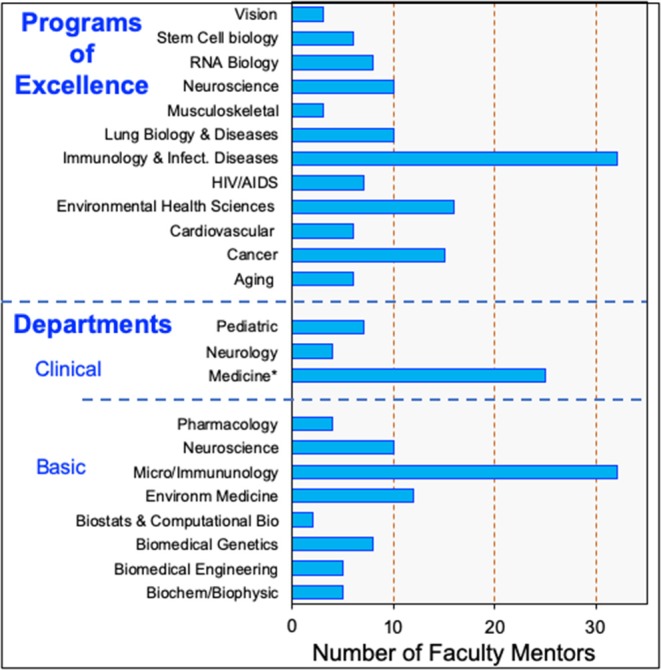
Research Expertise and Interconnectivity of UR-PREP Training Faculty. Number of faculty with research projects within 13 of the 14 URSMD Programs of Excellence, and with departmental primary/secondary appointments. *Medicine includes multiple departments, only Neurology and Pediatric with more than 1 training faculty are mentioned.

**Figure 3:**
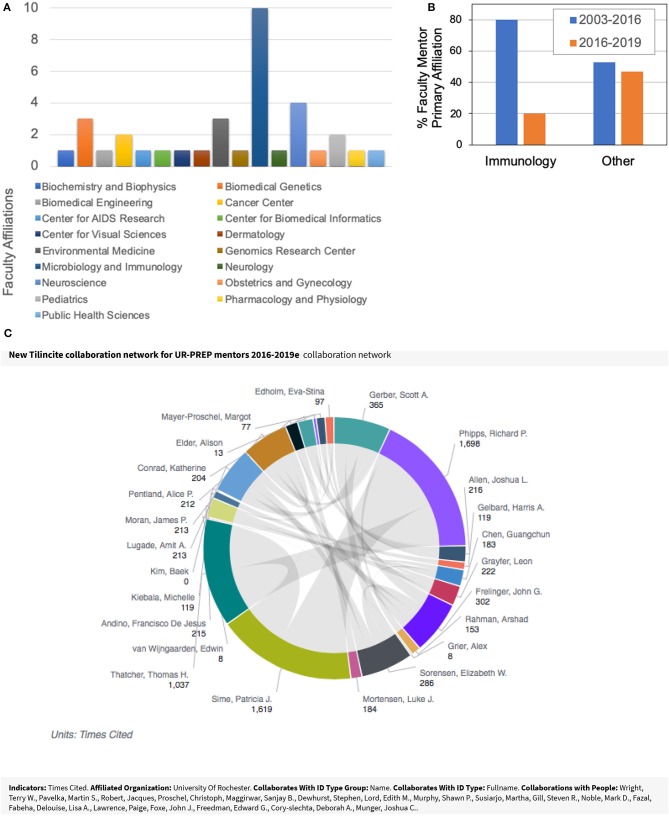
UR-PREP Interdisciplinary Network **(A)**. Graphical representation of faculty appointments of our UR-PREP mentors who have trained or are currently training scholars (2016–2019). **(B)** Graphical representation of our UR-PREP scholars who trained with faculty mentors holding primary appointments in Immunology compared to primary affiliations in other research fields from 2003 to 2016 and 2016 to 2019. **(C)** Interdisciplinary network of our UR-PREP mentors. Shown is a visual representation of the expanded collaborations of our mentors over the last 3 years. The middle gray area is the centralized hub of the mentors with a web of affiliated collaborators and co-authors (Data collected using Web of Science and ORCID identifiers at the URSMD).

Immunology is a research field with broad reach and high potential for fostering collaboration and scientific innovation. The URSMD recognizes that our learner populations seek opportunities to bridge these immunological reaches and encourages interdisciplinary research, which allow our learners to explore and apply their research perspectives. As such, UR-PREP has extended its available research training opportunities to our URGs beyond the Department of Microbiology and Immunology. Via communicating directly with Chairpersons from all of our research departments and centers across the URSMD, we have identified researchers with sufficient mentoring and training history to include in our UR-PREP mentor pool. We specifically include motivated junior faculty who bring fresh perspectives to our program. We were also mindful of addressing diversity with regards to gender equality and race/ethnicity. This outreach led us to extend our scientific research fields from Microbiology/Immunology (inclusive of ~25 mentors) to as many as 15 scientific fields including ~60 mentors (Figure 2). Our faculty mentors now encompass a range of research disciplines that can connect with immunology including cancer, cardiovascular disease, HIV/AIDS, respiratory diseases, stem cell biology, neurodegenerative diseases, toxicology, and RNA biology. Furthermore, these training opportunities comprise both fundamental and clinical research areas that are related to health disparities (e.g., HIV/AIDS), which are likely of interest to our scholars. The attractiveness of these options is evidenced by the wide range of academic appointments and affiliations to research departments and centers of mentors who have trained our UR-PREP scholars over the past 3 years (2016–2019; Figure 3A). For purposes of comparison, Figure 3B shows that from 2003–2019, 80% of our scholars trained with faculty who had primary appointments in Immunology compared to only 20% in other research fields. In the last three recruited cohorts, however, there has been a more even distribution of scholars between Immunology faculty (53%) and other disciplines (43%). This branching from the Immunology hub is important as it extends the research network of collaborators and fosters interaction between our scholars and faculty beyond their mentors (Figure 3C).

#### Diversifying Our URSMD Graduate Programs

It is noteworthy that as a result of broadening our research areas we witnessed an unintended but positive effect on retaining PREP scholars in our graduate programs. Prior to extending beyond immunology, our UR-PREP scholars were competing with each other for the same graduate student placements in the Immunology graduate program at the URSMD. Many of our scholars were also competing for placement at other graduate schools within the same immunology focused training programs. By broadening our UR-PREP training opportunities, our scholars are now applying to more diverse programs at the URSMD. Indeed, in the past 3 years UR-PREP scholars have applied to Immunology, Cell Biology of Disease, Toxicology, Translational Biomedical Sciences, Neuroscience, and Biomedical Genetics. Consequently, our UR-PREP scholars are remaining at the URSMD for their graduate studies, which has contributed to an overall increase in the number of URG graduate trainees at the URSMD from 10.6% in 2016 to 17.8% in 2019. Importantly, UR-PREP scholars constitute the majority (~10%) of this increase in URG graduate trainees. Interestingly, among the URGs matriculated into our graduate school, 27.5% (a substantial increase from 13.3% in 2016) have been awarded training fellowships and/or individual fellowships/awards, including some of our UR-PREP scholars. Collectively, this is evidence that our pipeline program critically contributes to diversifying our student population.

Table 2 shows the graduate programs into which 7 former UR-PREP scholars have matriculated and are currently still training at the URSMD. This also provides a snapshot of research areas pursued by each scholar as part of their training. In line with our goal to broaden research disciplines, none of the current students listed in Table 2 are in the Immunology graduate program, although 6 out of the 7 are conducting research that includes immunology aspects and involves mentorship within the Department of Microbiology and Immunology. We surveyed these students to inquire why they choose URSMD for their training. The following are representative testimonials of their responses:

**Table 2:** Currently matriculated UR-PREP scholars in URSMD.

**Scholar year**	**URSMD graduate program**	**Research project summary**
2015–2016	Cell Biology of Disease	Elucidating a potential mechanism of action of Bacillus Calmette-Guérin (BCG) immunotherapy for non-muscle invasive bladder cancer
2016–2017	Translational Biomedical Sciences	Applied immunology and microbiology in the clinical setting, including atopic and inflammatory diseases
2016–2017	Translational Biomedical Sciences	High-throughput approach to profiling the differences in antigen presentation between young and older adults to define drug repurposing targets for improving vaccine efficacy
2016–2017	Translational Biomedical Sciences	Prenatal immunological and stress factors associated with childhood development
2017–2018	Neuroscience	Molecular and Signaling Mechanisms of Synaptic Plasticity in Memory Formation and Mental Health
2018–2019	Translational Biomedical Sciences	The impact that the oral microbiome has on the outcome of disease. Specifically, how different environmental factors play a role in influencing microbiome composition and how this can be used as a tool to predict or prevent disease outcomes
2018–2019	Cell Biology of Disease	Understand the co-evolutionary relationships between the structure of selected molecules and their functions in innate and adaptive immunity against tumors and viruses using the frog *Xenopus laevis* as animal model

“Welcoming environment”

“Collaborative opportunities”

“[Translational Biomedical Sciences graduate program] provided a unique way to combine bench and clinical science.”

“[I] had a great experience in lab/PREP, the mentorship I received gave me confidence that the type of support needed in a PhD program would be available.”

Thus, the matriculation of our UR-PREP scholars into programs beyond immunology is significantly contributing to increasing diversity across the institution and reducing the biases that have long been associated with the ability of minority biomedical researchers to be successful (10–12).

### Curriculum Modifications

We recognize the necessity of modifying our educational curriculum to supplement the broadened research experiences of our program. Therefore, we have taken several steps in the past 3 years to enhance our educational programming. These steps, described herein, are designed to provide high quality laboratory-based research education, are have been adjusted to the specific needs of URGs.

Evidence has shown that learners from URGs have unique experiences and attitudes regarding access to research, academic, and professional development opportunities (12–15). Thus, URGs do not necessarily enter graduate school with the same academic and skill proficiencies as their non-URG counterparts (5). These decreased proficiencies are often the primary factor in determining a scholar's success in academic programs (16). UR-PREP addresses these concerns by providing URGs an opportunity to develop research and academic skills as well as provide better psychological and professional support.

#### Immediate Readiness and Team-Building—Basic Skill Workshop

In 2016 we implemented an intensive 2-week basic laboratory and soft skills workshop at the beginning of the UR-PREP year in conjunction with the URSMD Life Sciences Learning Center co-directed by Dina Markowitz, PhD, and Danielle Alcena, PhD, who is a former UR-PREP scholar. This workshop is designed to introduce common laboratory principles and techniques (e.g., molarity, pH, dilution, cell culture, statistics, etc.) and soft skills (e.g., oral presentation, abstract writing, conflict management, etc.). We also seek to identify potential challenges by administering short quizzes and more extensive take-home exercises, allowing us to assess progress and areas where additional training is needed. Importantly, this 2-week experience fosters a group dynamic and builds a team spirit that has been tremendously helpful for our PREP scholars during the challenging training period. Since our scholars disseminate into labs across the URSMD from our Immunology hub, we have to ensure that they are prepared to enter their research niches with confidence and a sense of belonging. While only in its third year, we have received positive feedback about our workshop with regards to the scholars' ability to quickly integrate into their laboratory settings and begin active research projects (selected testimonials below).

“The workshops put everyone on an even playing field and assures that they at least have the fundamental techniques down before beginning work on their independent research projects.” (*2017–2018 scholar and current URSMD Neuroscience graduate student*)“[I] think having a period at the beginning where PREP students can become accustomed to the school and each other is very useful.” (*2018–2019 scholar and current URSMD Translational Biomedical Sciences graduate student*)

“[The student workshop leader] was great! Overall, the training was a good review before getting started in the lab.” *(2019–2020 current scholar)*

#### Academic Autonomy

All of our UR-PREP scholars are required to take a graduate level course, Ethics and Professional Integrity, and a student research seminar to prepare them for the academic rigors of graduate school. Given the history of our academic and research roots in Immunology, our UR-PREP cohorts have primarily enrolled in the basic Immunology course and related student seminar. However, with the broadening of our research program, our scholars can now select courses that are relevant to their research goals. Recent enrollment is as diverse as: Cell Biology of Human Disease; Cellular Neuroscience; Foundations in Modern Biology; Genomics and Systems Biology; Microbial Pathogenesis; Neurons, Circuits and Systems. Student seminars have included: Current Topics in Experimental Pathology, Genetics, Microbiology, Immunology, and Neuroscience. By gaining academic enrichment outside of Immunology, our UR-PREP scholars are interfacing with more faculty and students, as all of our courses are typically team taught by faculty with expertise in certain subject areas with the help of PhD student and postdoctoral teaching assistants. Thus, our scholars are benefitting from selecting courses that are best for their growth, as well as gaining new academic knowledge that will be beneficial to their scientific careers.

Importantly, aside from taking course related to their research interests, our optimized program also requires our UR-PREP scholars to take a professional development course or attend URSMD sponsored workshops to supplement their training. There is evidence to support that coaching URGs in how best to reach their goals outside of laboratory skill development is integral to their success (8, 14, 17). As such, our scholars are advised to select from many courses and workshops offered by the URMSD, Center for Professional Development, and UR Broadening Experiences in Scientific Training that suit their professional growth. Within the past 3 years our scholars have enrolled in Leadership and Management for Scientists, Scientific Communication for Broad Audiences, Scientific Writing in Research, and Drug Discovery, and have attended workshops including Resume and Curriculum Vitae (CV) Writing, Interviewing, Grant Writing, and Manuscript Writing. Furthermore, our scholars met with our Director of Career Services within their first 2 months of the UR-PREP year to build their CVs, establish a professional LinkedIn social media presence, and begin their Individual Development Plans.

### Team Approach to Broadening Leadership Perspectives Beyond Immunology

**Figure 4:**
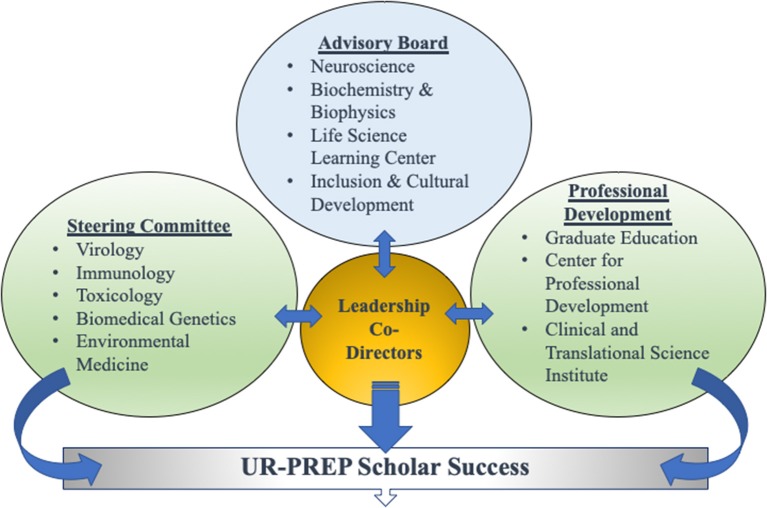
Team Leadership of UR-PREP. The Co-Directors are informed by a large group of faculty, students, and postdoctoral trainees who reside in a vast array of scientific and professional disciplines, all designed to promote UR-PREP scholar success.

Another key aspect of our extended URG training program is its team leadership concept. We have implemented a co-director team leadership approach grounded in complementary expertise by two directors: Research Development (Jacques Robert, PhD) and Professional and Academic Mentorship (Elaine Smolock, PhD). While the research development director more specifically oversees research components of the training (e.g., workshop organization, interface with research mentors, scientific contents of scholar projects, conference attendance), the professional/academic director focuses on communication skills and professional preparation (e.g., writing, communication, scholarship, academic progress, course advising). This dual and integrated leadership is enriched by a team-based environment providing advice and feedback from an Advisory Committee, Steering Committee, and Professional Development Group. This model is specifically designed to include faculty, students, postdoctoral appointees, counselors, and professionals from many scientific disciplines, expertise, and backgrounds (Figure 4). This insures a system of guidance, assistance, and support that is broad in perspective and appropriate to our UR-PREP scholars' research and career goals. This cohesive team-based program is directly beneficial to the scholars in that they have ample options to feel individual ownership of their research experience by meeting with any of the team members, allowing them to feel highly connected to the larger URSMD community.

### Heightening Scholar-Community Integration and Outreach

A crucial aspect to the success of UR-PREP is fostering a strong sense of community and inclusiveness among our scholars within the URSMD. Indeed, learners from URGs can struggle with finding their academic identify (4). Admittedly, the long-standing Immunology centricity of our UR-PREP was successful in building unity and cohesiveness among our UR-PREP cohorts; however, that model was less successful in integrating our scholars within the greater student body. With the recent broadening of UR-PREP beyond immunology, we have taken key steps to better integrate our scholars and retain the cohesiveness of the group, with the goal of fostering a research identity and a sense of belonging.

#### PREP Council

We initiated a PREP Council in 2016. This organization includes the current UR-PREP scholars, a postdoctoral fellow, and/or senior PhD student who is from a URG with interest in education. This is a critical aspect of our training program, as there is evidence that near-peer URG mentors build inclusivity and awareness of the unique needs of trainees from URGs (8). It is also an opportunity for our current graduate students and postdoctoral appointees to develop their own cultural agency (18).

The council hosts an annual symposium featuring previous UR-PREP scholars who return to the URSMD to discuss their post UR-PREP research and career experiences in both academic and non-academic environments (e.g., industry, government). In addition, the council organizes an annual seminar given by a renowned extramural guest speaker. The scholars are encouraged to invite speakers in the scientific disciplines of their interests to promote an extended view beyond immunology and provide perspective about scientific careers. The Council recently connected with the URSMD Alliance for Diversity in Science and Engineering (ADSE) group that hosted Avery August, PhD (HHMI Professor of Microbiology and Immunology at Cornell University) who spoke about his experiences as a URG biomedical researcher. These events are widely advertised across the institution to raise attention regarding the involvement of our URG learners.

#### Cultural Capital, Awareness, and Advocacy

As mentioned above, establishing cultural capital among students from URGs is crucial for their success (4, 19). Notably, this capital increases graduate school readiness (16). We have recently made efforts to better help our scholars establish identity and integrate into the larger scientific community at our institution. We have taken steps to connect the scholars with the URSMD Office of Diversity and Inclusion. Specifically, our UR-PREP scholars meet at least three times per year with our Diversity Officer (John Cullen, PhD). These meetings happen in the absence of the co-directors to allow the UR-PREP scholars opportunities to advocate for themselves and freely discuss any concerns. This innovative approach has been instituted to promote better understanding of our UR-PREP scholar needs and teach them how to discuss the value of their diverse perspectives in academia and biomedical research broadly.

## Discussion

The implementation of our UR-PREP to include a more diversified biomedical research experience extending beyond immunology and a more integrated team- and inclusion-based leadership approach has only been ongoing for 3 years. However, there is tangible evidence of success exemplified by the percentage of UR-PREP scholars entering graduate school in areas beyond Immunology, as well as a change in attitude and perception toward URGs within the institution. Faculty members and students from the different departments and centers now have increased opportunities to interact with our UR-PREP scholars, which has beneficial effects in easing misconceptions and minimizing unconscious bias toward URGs.

Given the relative short time (3 years/3 cohorts), our data are preliminary, but suggest steady increase in success (e.g., broadening of research opportunities, scholar matriculation into graduate school, inclusivity, etc.). Our analysis has also revealed a few areas that would merit further modification. A first concern is that broadening the research opportunities away from the Immunology hub could potentially reduce a sense of cohort. To thwart this, we have developed weekly UR-PREP courses where we promote group-based discussions and preparation for graduate school. These regular meeting are a good setting to evaluate progress and unexpected challenges or difficulties encountered by a particular scholar or by the whole group. Our active UR-PREP Council should also counteract potential isolation of our scholars in different areas of our institution. Likewise, together with our Office of Diversity and Inclusion and the ADSE student group, we are actively promoting social outings and outreach opportunities that are intended for team-building. As an example, a recent partnership with the Graduate Women in Science leadership, Catherine Ovitt, PhD, our UR-PREP scholars visited a local Rochester public school and discussed their experiences in STEM. Subsequently, our UR-PREP Council organized and hosted an on-site visit of high school students who are interested in pursuing STEM.

A second potential challenge in our training model is that the broadening of courses offered to our scholars may lessen ability for study groups, with the risk for some scholars to be insufficiently prepared and ultimately fail a class. To address this, we established a tight communication system with the course directors so that we can rapidly be aware of any scholar who is struggling, find tutors to supplement in-class learning, and/or provide additional study sessions when necessary.

In summary, we are successfully diversifying and enriching the experiences of URGs by extending beyond basic Immunology training. Our restructured and broadening of our UR-PREP pipeline is now more inclusive of our scholars' interests and unique academic and professional goals. However, there is still a paucity of URGs in faculty positions at academic institutions and a need to provide checkpoints to insure continued success (6). Furthermore, data of the National Institutes of Health tracked from 2009 to 2016 reveal a need to prioritize funding investments and support of URG early-stage and new investigators (7). Together these findings emphasize the importance of pipeline programs such as PREP. As a future direction of our UR-PREP, we will be mindful of the challenges and circumstances that continue to face our PREP scholars after they matriculate and pursue careers in biomedical research. In line with NIH-funded National Research Mentoring Network models (20), we are currently planning long-term mentoring plans to provide support throughout career development. The goal is to improve retention of our scholars in biomedical research so that there is better representation of URGs at the faculty level, hopefully someday minimizing the need for these pipelines programs.

## Data Availability Statement

The datasets generated for this study are available on request to the corresponding author.

## Author Contributions

ES and JR have been involved equally in the conception and writing this article.

### Conflict of Interest

The authors declare that the research was conducted in the absence of any commercial or financial relationships that could be construed as a potential conflict of interest.
